# Cognitive fitness modulates gender differences in sleep and mental health among competitive athletes under chronic stress

**DOI:** 10.3389/fphys.2023.1118822

**Published:** 2023-03-08

**Authors:** Luis Mascaro, Sean P. A. Drummond, Josh Leota, Johanna M. Boardman, Daniel Hoffman, Shantha M. W. Rajaratnam, Eugene Aidman, Elise R. Facer-Childs

**Affiliations:** ^1^ Turner Institute for Brain and Mental Health, Monash University, Melbourne, VIC, Australia; ^2^ School of Psychological Sciences, Monash University, Melbourne, VIC, Australia; ^3^ St Kilda Football Club, Australian Football League, Melbourne, VIC, Australia; ^4^ Defence Science & Technology Group, Edinburgh, SA, Australia; ^5^ School of Biomedical Sciences & Pharmacy, University of Newcastle, Newcastle, NSW, Australia; ^6^ Danny Frawley Centre for Health and Wellbeing, Melbourne, VIC, Australia

**Keywords:** sport, self-control, impulsivity, depression, anxiety, intolerance of uncertainty, sleep, pandemic

## Abstract

**Objectives:** Mental fitness is increasingly considered a key component of an athlete’s competitive arsenal. Active domains of mental fitness include cognitive fitness, sleep, and mental health; and these domains can differ between men and women athletes. Our study investigated the associations of cognitive fitness and gender to sleep and mental health, and the interaction between cognitive fitness and gender on sleep and mental health, in competitive athletes during the COVID-19 pandemic.

**Methods:** 82 athletes competing at levels from regional/state to international (49% women, *M*-age = 23.3 years) completed measures of self-control, intolerance of uncertainty, and impulsivity (together representing constructs of cognitive fitness), items about sleep (total sleep time, sleep latency, and mid-sleep time on free days) and a measure of mental health (depression, anxiety, and stress).

**Results:** Women athletes reported lower self-control, higher intolerance of uncertainty, and higher positive urgency impulsivity compared with men athletes. Women reported sleeping later, but this gender difference disappeared after controlling for cognitive fitness. Women athletes—after controlling for cognitive fitness—reported higher depression, anxiety, and stress. Across genders, higher self-control was associated with lower depression, and lower intolerance of uncertainty was associated with lower anxiety. Higher sensation seeking was associated with lower depression and stress, and higher premeditation was associated with greater total sleep time and anxiety. Higher perseverance was associated with higher depression for men—but not women—athletes.

**Conclusion:** Women athletes in our sample reported poorer cognitive fitness and mental health compared to men athletes. Most cognitive fitness factors protected competitive athletes under chronic stress, but some exposed them to poorer mental health. Future work should examine the sources of gender differences. Our findings suggest a need to develop tailored interventions aimed at improving athlete wellbeing, with a particular focus on women athletes.

## 1 Introduction

The ability to perform under high-pressure and stressful conditions have far-reaching implications in several mission-critical occupations, including the military, first responders, and competitive athletes. These professionals are motivated to seek strategies and modifiable factors to prepare for high-pressure situations. For example, athletes regularly engage coping skills to protect their performance against the influence of potentially acute stressors such as crowd pressure, referee decisions, and skill errors (Rice et al., 2016). Chronic stressors—both within and outside of their job—also impact performance and wellbeing. For example, frequent and long-distance air travel is known to disrupt sleep and circadian rhythms which can lead to chronic jet lag and impair performance (Fowler et al., 2017; Leota et al., 2022). Similarly, after a long-term injury, athletes report mental health symptoms and a low level of psychological readiness to return to competition at their best despite full physical recovery (Ruddock-Hudson et al., 2014). Researchers and practitioners increasingly recommend addressing potentially modifiable domains of functioning, such as cognition, sleep, and mental health, to help athletes build resilience to and recover from both acute and chronic stressors and improve their performance (Fletcher and Sarkar, 2016; Reardon et al., 2019; Walsh et al., 2021).

The coronavirus-2019 (COVID-19) pandemic is a quintessential chronic stressor, characterized by unprecedented levels of uncertainty and lifestyle changes (Holmes et al., 2020) associated with socio-economic consequences (Nicola et al., 2020) and physical health deterioration (Woods et al., 2020). In the general population, about one in three individuals experienced poor sleep or mental health during the pandemic (Jahrami et al., 2021; Wu et al., 2021). During this time, lifestyle changes for athletes have included stay-at-home orders, social isolation, cancelled competitions, disrupted training regimes, and threats to income and wellbeing (Woodford and Bussey, 2021). Isolation from the sport, coaching team, and fans was identified pre-pandemic as a risk factor for psychological distress in athletes (Reardon et al., 2019). Upon the onset of the pandemic, these concerns were realized in the form of various sleep and mental health changes (Pillay et al., 2020; Facer-Childs et al., 2021). Athletes have typically been characterized as more resilient to stressors than non-athletes (Laborde et al., 2016). However, the COVID-19 pandemic revealed that even among athletes there exists high variation in resilience and outcomes (Balcombe and De Leo, 2021). A closer examination of inter-individual differences in athletes is warranted to determine which traits may be accounting for differences in sleep and mental health during this chronic stressor and to identify potential areas for intervention.

General cognitive functioning (e.g., self-control), sustained readiness (e.g., tolerance of uncertainty), and decision-making (e.g., impulsivity)—together constituting ingredients of cognitive fitness (Aidman, 2020)—are known for their capacity to protect against poor sleep and mental health deterioration (Kashdan and Rottenberg, 2010; Schmidt et al., 2010; Hasegawa et al., 2018; Lauriola et al., 2019). Additionally, gender can also influence sleep and mental health. During the pandemic, women experienced more mental health problems and lower psychological wellbeing compared to men (Daly et al., 2020; Vindegaard and Benros, 2020). In athletes, women also reported worse mental health outcomes compared to men during the pandemic (FIFPRO World Players’ Union, 2020; Jurecka et al., 2021; Di Fronso et al., 2022), though athlete gender differences regarding sleep during this time are less clear (e.g., Jaenes Sanchez et al., 2021; Lorenzo Calvo et al., 2021).

Considering gender differences as an important influence on outcomes during chronic stress is consistent with calls for more research with women athletes (Cowley et al., 2021). Furthermore, some studies suggest that women athletes report lower resilience (Özdemir, 2019; Blanco-García et al., 2021). As such, the possible interactive effects of cognitive fitness (Aidman, 2020) and gender on sleep and mental health outcomes could have implications for intervention strategies with athletes. Indeed, the ingredients of cognitive fitness studied in the current paper have the potential to be modified to improve performance and wellbeing (Balk and Englert, 2020; Aidman et al., 2022; Flood and Keegan, 2022; Albertella et al., 2023). The COVID-19 pandemic provided a naturalistic example of a global chronic stressor for us to explore the possible associations between cognitive fitness and gender with wellbeing under real-world conditions of high vulnerability.

The aims of the present exploratory study were to: 1) investigate gender differences in cognitive fitness (self-control, intolerance of uncertainty, and impulsivity), sleep (duration, timing, and onset latency), and mental health (depression, anxiety, and stress) in competitive athletes; 2) investigate the association between cognitive fitness and sleep and mental health outcomes; and 3) investigate the interactive effects of gender and cognitive fitness on sleep and mental health.

## 2 Materials and methods

### 2.1 Participants

Eligibility criteria required participants to be at least 18 years of age, a regional/state, national or international level competitive athlete, reside in Australia, and not be a full-time shift worker. Participants were recruited *via* social media and contacts within sporting clubs. The final sample size of eligible participants was 82 competitive athletes (49% women). Demographic data were collected *via* self-report, including self-identified gender. While not the focus of the paper, we note the distinction in terminology between sex (based on physiology, e.g., male/female) and gender (a social construct, e.g., men/women). Thus, based on the question used to collect data here, we will refer to men and women competitive athletes for our sample (Madsen et al., 2017). The average age of the sample was 23.27 years (*SD* = 3.91). See Tables 1, 2 for further demographic information. The study was approved by the Monash University Human Research Ethics Committee.

**TABLE 1:** Demographic data for all athletes and split by gender.

	Whole sample (*N* = 82)	Men athletes (*n* = 42)	Women athletes (*n* = 40)	*p*-value
Age (years)	23 (18–35)	23.5 (18–32)	22 (18–35)	ns
Height (cm)	180.22 (±12.79)	189.00 (±9.16)	171.00 (±9.01)	**<.001**
Weight (kg)	76.11 (±13.18)	86.19 (±8.30)	65.53 (±7.99)	**<.001**

Age was non-normally distributed, so presented is Median (Range) and a pairwise comparison using the Mann-Whitney U test. Height and weight Means (±SD) and independent *t*-tests are presented. ns, not significant.

Significant *p*-values are indicated in bold.

**TABLE 2:** Breakdown of sporting and employment details.

Variable	*n*	%
Sport		
Australian football	63	76.8
Water sports^a^	7	8.5
Dance sports^b^	7	8.5
Basketball	4	4.9
Racket sports^c^	4	4.9
Athletics/running	3	3.7
Other	13	15.9
Highest competition standard		
International	5	6.1
National	68	82.9
Regional/state	9	11.0
Employment/student status		
Employed full-time	48	58.5
Employed part-time	15	18.3
Self-employed	2	2.4
Student full-time	17	20.7
Student part-time	9	11.0
Temporarily stood down (with pay)	1	1.2
Temporarily stood down (without pay)	6	7.3
Unemployed	2	2.4

^a^ Comprises of dragon boat racing, swimming, rowing, underwater hockey, and water polo.

^b^ Comprises of cheerleading, dance, and calisthenics.

^c^ Comprises of tennis, badminton, and squash.

Count and percentage for sport and employment exceed 100% due to multi-selections available.

### 2.2 Procedure

A questionnaire battery was administered electronically *via* Qualtrics to athletes in Victoria (*n* = 78, 95%) and New South Wales (*n* = 4, 5%), Australia. Informed consent was provided prior to completion. The sleep and mental health measures were administered during May 2020, at which time Victoria and New South Wales were under government-mandated stay-at-home orders (Czeisler et al., 2021). During this period, all sporting competitions were cancelled, and athletes could train alone at home or with one other person socially distanced outside the home.

The cognitive fitness measures were administered during August 2020, at which stage many sporting competitions had returned. Our measures of cognitive fitness have not had their test-retest reliability assessed within the context of a chronic stressor or pandemic, so we cannot rule out the possibility of some change across this period. However, despite the time delay and potential differences in lockdown conditions, the measured traits are considered generally stable (Buhr and Dugas, 2002; Anusic and Schimmack, 2016; Jordalen et al., 2018; Kaiser et al., 2018). In addition, these measures were administered after the peak of local COVID-19 cases and as cases were declining (Czeisler et al., 2021). Therefore, even if there were some state-like components to the measures, the scores obtained here were likely not influenced by the peak of psychosocial stress and, thus, may better reflect the trait-like levels of these constructs in our participants.

### 2.3 Measures

#### 2.3.1 Cognitive fitness

The Brief Self-Control Scale (BSCS; Tangney et al., (2004)) measures the discipline and restraint of behavior in response to changes in the environment. The scale consists of 13 items (e.g., “I am good at resisting temptation”) answered on a 5-point Likert scale from 1 (“not at all like me”) to 5 (“very much like me”). Higher total scores indicate greater self-control. The BSCS shows strong concurrent validity with other measures of behavioral control and strong predictive validity with several measures of achievement (Lindner et al., 2015). In the current athlete sample, the internal consistency was high (*α* = .85).

The Intolerance of Uncertainty Scale (IUS-12; Carleton et al., (2007)) measures the tendency of an individual to interpret ambiguous events as threatening. The scale consists of 12 items (e.g., “I cannot stand being taken by surprise”) answered on a 5-point Likert scale from 1 (“not at all characteristic of me”) to 5 (“entirely characteristic of me”). Higher total scores indicate greater *in*tolerance of uncertainty. The IUS-12 demonstrates strong internal consistency and strong concurrent validity with measures of anxiety and worry (Carleton et al., 2007). In the current athlete sample, the internal consistency was high (*α* = 0.92).

Impulsive behavior was measured using the short UPPS-P scale (S-UPPS-P; Dugré et al., 2019), which measures negative urgency (U), premeditation (P), perseverance (P), sensation seeking (S), and positive urgency (P). The two urgency scales refer to the tendency to act rashly when in a positive (positive urgency) or negative (negative urgency) mood; sensation seeking is the tendency to seek thrilling and novel experiences; perseverance refers to staying focused during long, boring, or difficult tasks; and premeditation refers to the consideration of the consequences of actions when deciding whether to act or not (Dugré et al., 2019). As an example, one item on the negative urgency subscale is “when I am upset I often act without thinking.” Each subscale consists of four items answered a 4-point Likert scale from 1 (“disagree strongly”) to 4 (“agree strongly”). Higher impulsivity is associated with higher negative urgency, positive urgency, and sensation seeking, and lower premeditation and perseverance. The five-factor structure of impulsivity has been validated, and the scale is reliable across genders (Dugré et al., 2019). In the current athlete sample, the internal consistency for all subscales were in the acceptable range (α’s 0.70–0.84).

#### 2.3.2 Sleep and mental health

The survey included one-item questions for total sleep time (TST; “hours I actually slept”) and sleep latency (SL; “minutes to fall asleep”) as retrospective self-reports for typical patterns over the past month. The wording of these items were adapted from the validated Pittsburgh Sleep Quality Index (Buysse et al., 1989) and have demonstrated adequate corrected item-total correlations (Beaudreau et al., 2012). One-item questions regarding typical sleep onset and offset times on training- and competition-free days over the past month were used to calculate mid-sleep time on free days (MSF; the midpoint between these values). The items assessing MSF were adapted from the validated Munich ChronoType Questionnaire (Roenneberg et al., 2003), have been shown to predict circadian timing (Kantermann and Burgess, 2017), and when assessed retrospectively in athletes may be more likely capture typical behavior compared to a smaller sample size of free days if attempting daily monitoring. We also used one-item measures (rather than daily diaries, objective measures, or the longer-length questionnaires) to reduce participant burden and increase scalability of the survey distribution.

The Depression Anxiety Stress Scales (DASS-21; Lovibond and Lovibond, (1995)) were used to measure self-reported mental health symptoms experienced over the past month. There were seven items per subscale (depression, anxiety, and stress). Items were answered on a 4-point Likert scale from 0 (“does not apply to me at all”) to 3 (“applied to me most of the time”). As an example, one item on the depression subscale is “I felt down-hearted and blue”. Higher total scores indicate worse mental health (i.e., higher on each construct). Strong validity and reliability of the DASS-21 have been demonstrated in non-clinical samples (Henry and Crawford, 2005), and in athlete samples during the COVID-19 pandemic (Vaughan et al., 2020). In the current athlete sample, the internal consistency for the depression (*α* = 0.91), anxiety (*α* = 0.77), and stress (*α* = 0.89) subscales were in the acceptable range.

### 2.4 Data analysis

Data analysis was completed using R Core Team, (2021), with statistical significance set at *p* < 0.05. Two men athletes had missing data on the sleep and mental health measures. To investigate gender differences on all measures, descriptive statistics and independent *t*-tests were used. A Welch correction was applied where homogeneity of variance was violated. Where at least one gender had a variable that was non-normally distributed, a Mann-Whitney U test was conducted instead. Cohen’s *d* was used to interpret effect sizes of the *t*-test: small, .20 ≤ *d* < 0.50; medium, .50 ≤ *d* < 0.80; large, *d* ≥ 0.80 (Cohen, 1992). Cohen’s *r* was used to interpret effect sizes of the Mann-Whitney U test: small, .10 ≤ *r* < 0.30; medium, .30 ≤ *r* < 0.50; large, *r* ≥ 0.50 (Cohen, 1988).

Correlations between all variables were computed to examine basic bivariate associations. Cohen’s *r* was used to interpret effect sizes of the correlations. To further investigate the association between cognitive fitness variables (as predictors) and sleep and mental health outcomes, a generalized linear model (GLM) for each outcome measure was applied. Across each model, gender was the consistent predictor variable, alongside select cognitive fitness variables. All cognitive fitness variables are theoretically justified to be included in the models in addition to gender (Aidman, 2020). However, our relatively small sample size precludes us from including all seven (BSCS, IUS, and 5 subscales of the S-UPPS-P) to not risk increasing the Type I error rate (Ioannidis, 2005). Limiting the number of predictors is also more likely to inform feasible and practical sports-science recommendations.

The glmnet package in R was used to select which cognitive fitness variables were to be included (Friedman et al., 2010). This method uses cross-validation to fit a proposed GLM *via* penalized maximum likelihood. Using the elastic net penalty, it returns predictor coefficients at the lambda value that gives the least mean cross-validated error. Variables with non-zero coefficients in the cross-validation were selected for the final GLMs. The default number of folds (10) was used to cross-validate variable selection. An arbitrary cut-off of non-zero coefficients returning in 75% of unique sets was used to determine variable selection. This method yielded the same variable selection as if the number of folds was equal to the sample size (a method which is reproducible), thereby providing informal support for the arbitrary cut-off and final models. The GLM family was dependent on the distribution of the outcome variable. The Gaussian distribution was used for TST and MSF. The models that were candidates for the negative binomial (SL, depression, anxiety, and stress outcomes) were confirmed *via* graphical inspection and tests of over-dispersion.

We were also interested in the potential interaction between gender and the cognitive fitness variables. In a new model, each GLM had an additional interaction term between gender and each cognitive fitness variable. Any significant interaction was examined using simple slopes to aid interpretation.

## 3 Results

### 3.1 Descriptive statistics and gender differences

**FIGURE 1:**
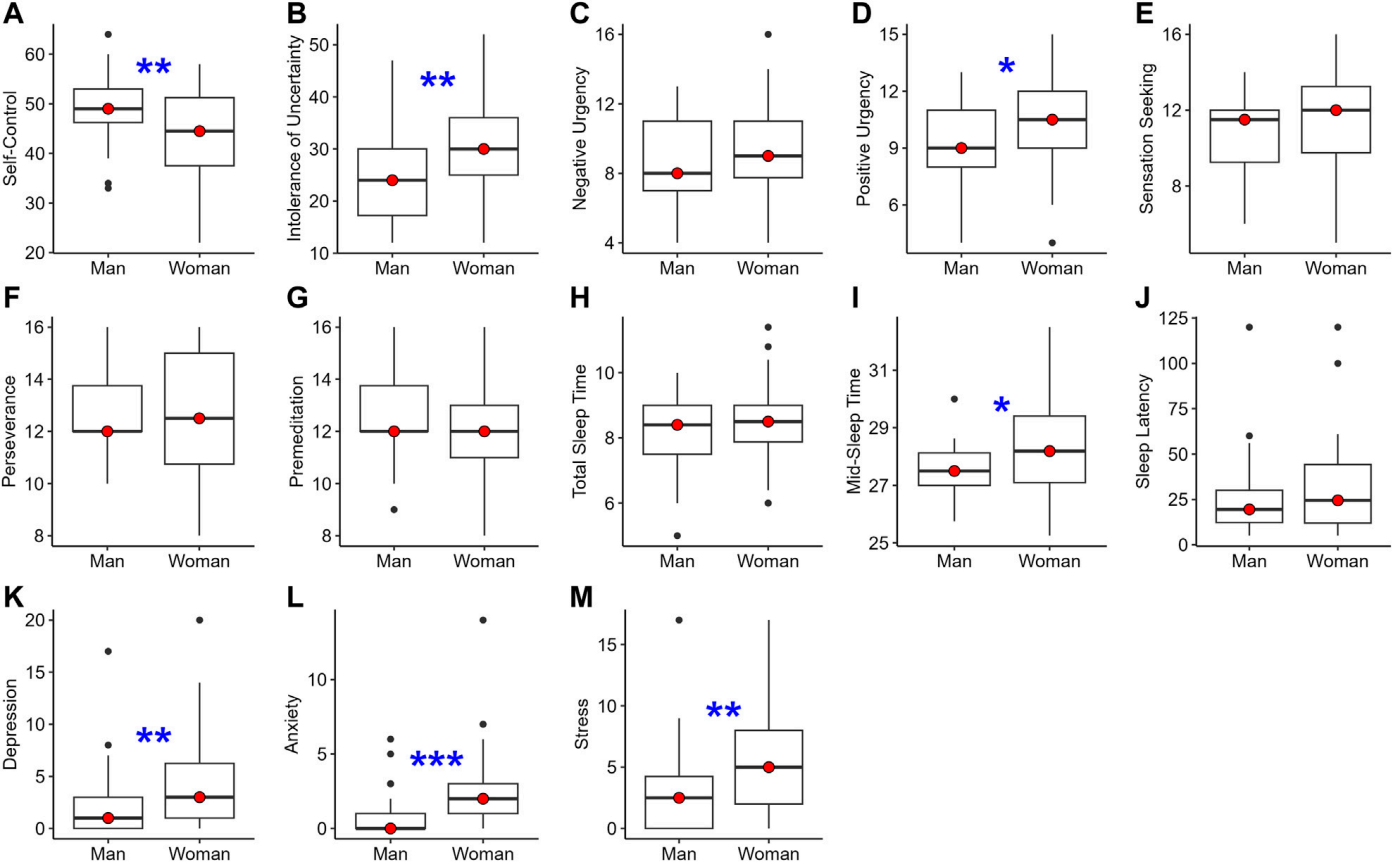
A visual representation of descriptive statistics for each measure (see each y-axis for Panels **(A–M)**). Separate box plots are plotted for men and women athletes. The thick line with the shaded circle in its center represents the median. The box represents the middle 50% of the data (i.e., from 25th to 75th percentile). The lines extended from the box go to the minimum and maximum of the data, excluding outliers (represented by dots). Stars between the boxes represent significant differences in the measure assessed by independent t-tests or Mann-Whitney U tests. *p < 0.05; **p < 0.01; ***p < 0.001.

Women athletes reported significantly lower self-control, higher intolerance of uncertainty, and higher positive urgency impulsivity. Women athletes reported sleeping later on free days by 43 min compared with men athletes. Women athletes also reported significantly greater depression, anxiety, and stress symptoms. All significant differences found were of a medium effect size (Table 3; Figure 1).

**TABLE 3:** Descriptive statistics for cognitive fitness, sleep, and mental health, and pairwise gender comparisons.

	Whole sample	Men athletes	Women athletes	|*d*| or |*r*|
Self-control^a^	46.40 (8.45)	49.29 (6.44)**	43.38 (9.29)	0.74
Intolerance of uncertainty	27.74 (9.33)	24.74 (9.37)	30.90 (8.27)**	0.70
Negative urgency	8.96 (2.55)	8.74 (2.29)	9.20 (2.81)	ns
Positive urgency	9.80 (2.35)	9.19 (2.18)	10.45 (2.39)*	0.55
Sensation seeking^a^	11.21 (2.38)	10.95 (1.97)	11.47 (2.74)	ns
Perseverance^a^	12.66 (2.16)	12.86 (1.75)	12.45 (2.52)	ns
Premeditation	12.40 (1.88)	12.62 (1.78)	12.18 (1.99)	ns
TST (hours)	8.26 (1.20)	8.13 (1.06)	8.40 (1.32)	ns
MSF^a^ (clock time ± h:mm)	03:56 (1:15)	03:35 (0:48)	04:18 (1:30)*	0.59
SL^b^ (mins)	20 (5–120)	19.50 (5–120)	24.50 (5–120)	ns
Depression^b^	2 (0–20)	1 (0–17)	3 (0–20)**	0.34
Anxiety^b^	1 (0–14)	0 (0–6)	2 (0–14)***	0.40
Stress^b^	3.5 (0–17)	2.5 (0–17)	5 (0–17)**	0.31

^a^ Welch correction applied t-test.

^b^ Non-normally distributed variable.

For normally distributed variables, presented are *M* (*SD*), independent t-tests, and Cohen’s *d* effect size. For non-normally distributed variables, presented are Median (range), Mann-Whitney U tests, and Cohen’s *r* effect size.

* ***p*** < 0.05; **p < 0.01; ***p < 0.001.

TST, total sleep time; MSF, mid-sleep time on free days; SL, sleep latency; ns, not significant.

Significant *p*-values are indicated in bold.

### 3.2 Relationship between cognitive fitness and sleep and mental health

Significant bivariate correlations were observed between several measures (Table 4; Supplementary Figure S1). Associations of at least a medium effect included: i) greater self-control correlated with earlier MSF, lower depression, and lower anxiety; ii) lower intolerance of uncertainty correlated with lower depression and anxiety; and iii) lower negative urgency correlated with lower depression and stress.

**TABLE 4:** Correlation matrix between cognitive fitness, sleep, and mental health.

	TST	MSF	SL^a^	Depression^a^	Anxiety^a^	Stress^a^
Self-control	0.05	−0.32**	−0.13	−0.47***	−0.33**	−0.28*
Intolerance of uncertainty	0.13	0.29**	0.25*	0.40***	0.44***	0.26*
Negative urgency	−0.04	0.11	0.03	0.31**	0.20	0.38***
Positive urgency	0.01	0.11	−0.06	0.19	0.29**	0.23*
Sensation seeking	0.15	−0.17	−0.14	−0.15	−0.09	−0.28*
Perseverance	0.11	−0.24*	−0.14	−0.05	−0.03	−0.11
Premeditation	0.25*	0.01	−0.02	−0.02	0.11	−0.16

^a^ Spearman’s rho used for correlations with non-normally distributed variables.

*N* = 80 complete observations only.

TST, total sleep time; MSF, mid-sleep time on free days; SL, sleep latency.

*p < 0.05; **p < 0.01; ***p < 0.001.

Significant *p*-values of at least medium effect are indicated in bold.

Results of the cross-validation regarding the predictors (in addition to gender) most likely to yield non-zero coefficients can be observed by the blank and non-blank cells in Table 5. Very little multicollinearity between predictors was observed across all GLMs (variance inflation factors around 1). All models, except for SL, *via* a likelihood ratio test were found to be significantly better predictors of the outcome than an intercept-only model.

**TABLE 5:** Generalized linear model output for the prediction of sleep and mental health.

Outcome variable	TST	MSF	SL^a^	Depression^a^	Anxiety^a^	Stress^a^
Predictor variable						
Gender (=Man)	0.84	0.78	0.87	0.77*	0.72*	0.78*
Self-control		0.80		0.59***	0.78	0.91
Intolerance of uncertainty		1.14		1.23	1.40*	1.05
Negative urgency						1.14
Positive urgency						1.09
Sensation seeking		0.81		0.77*		0.74**
Perseverance		0.90		1.39*		
Premeditation	1.38*			1.02	1.30*	
Model fit statistics						
McFadden Pseudo-R^2^	0.03	0.07	0.00	0.08	0.10	0.04
Log likelihood	−123.83	−121.97	−338.13	−170.16	−126.09	−193.29
Likelihood ratio test (*p*)	0**.03**	**0.004**	0.10	**<0.001**	**<0.001**	**0.006**

^a^ Negative binomial distribution used instead of Gaussian. Blank cells for predictor variables indicate variable not included in model after cross-validation screening. Model fit statistics between models cannot be interpreted due to different outcome measures. Likelihood ratio tests against an intercept-only model.

Presented for predictor variables are exponentiated standardized coefficients.

*p < 0.05; **p < 0.01; ***p < 0.001.

TST, total sleep time; MSF, mid-sleep time on free days; SL, sleep latency.

Significant *p*-values are indicated in bold.

Exponentiated standardized coefficients in Table 5 represent incident ratios. To interpret these findings, for each standard deviation increase in a cognitive fitness variable (or, being a man athlete) the predicted value in an outcome will multiply by the given coefficient after accounting for other variables in the model. For example, being a man athlete is associated with a score 23% lower (or 0.77 times) for DASS-21 depression relative to women. Further, a standard deviation increase in intolerance of uncertainty is associated with a score 40% higher (or 1.40 times) for anxiety.

Previously identified gender differences in depression, anxiety, and stress maintained after controlling for cognitive fitness. Mid-sleep time on free days was no longer different between men and women athletes after controlling for cognitive fitness. Regarding cognitive fitness: i) greater self-control was associated with lower depression; ii) greater intolerance of uncertainty was associated with greater anxiety; iii) greater sensation seeking impulsivity was associated with lower depression and stress; and iv) greater premeditation was associated with greater TST and anxiety.

Greater perseverance was associated with greater depression, however follow-up analyses including interaction terms revealed a gender by perseverance interaction. Simple slope analyses revealed a significant positive relationship between perseverance and depression among men athletes (*p* < .001). There was no significant relationship between perseverance and depression among women athletes (*p* = 0.92). No other interaction terms were significant.

## 4 Discussion

The current study aimed to examine the influence of gender and cognitive fitness on sleep and mental health outcomes in competitive athletes during the COVID-19 pandemic. We found that women athletes reported traits of lower self-control, higher intolerance of uncertainty, and higher positive urgency impulsivity. After controlling for cognitive fitness, no gender differences were found in sleep outcomes, and women athletes reported poorer mental health (depression, anxiety, and stress). After controlling for gender, three findings indicated self-reported greater levels of cognitive fitness were associated with more positive mental health and better sleep outcomes. Specifically, higher self-control predicted lower depression, lower intolerance of uncertainty predicted lower anxiety, and higher premeditation predicted a greater total sleep time. However, four findings indicated self-reported greater levels of cognitive fitness were associated with poorer outcomes. Specifically, lower sensation seeking impulsivity predicted higher depression and stress, higher premeditation predicted higher anxiety, and, for men only, higher perseverance predicted higher depression. Thus, some cognitive factors appear to protect competitive athletes against poor mental health or sleep in the face of a chronic stressor, while others are associated with poorer outcomes.

In our sample of competitive athletes, women reported lower self-control than men. This aligns with previous athlete studies conducted pre- and mid-pandemic (Dumciene and Sipaviciene, 2021; Martínez-Patiño et al., 2021). However, women reporting lower self-control may in fact reflect gender differences in beliefs, rather than the neurocognitive capacity, of behavioral inhibition (De Ridder et al., 2012). Thus, further research is needed to elucidate whether patterns of self-reporting reflect real-life behavioral vulnerabilities for women athletes.

We also found that women athletes reported higher intolerance of uncertainty than men athletes. This contrasts a finding of no gender difference in a group of athletes during the pandemic (Casali et al., 2021). However, this may be explained by how gender differences in intolerance of uncertainty (with women typically reporting greater levels) tend to enlarge if mental health pathology decreases (McEvoy et al., 2019). Indeed, exploratory analyses revealed our average DASS-21 total score to be lower than that reported by Casali et al. (2021), indicating intolerance of uncertainty as a potential intervention target even in the absence of known mental health challenges.

Competitive women athletes self-reported greater positive urgency impulsivity which was in contrast to pre-pandemic studies reporting no gender difference (Holfelder et al., 2020; Gabrys and Wontorczyk, 2022). However, gender differences in urgency have been reviewed as unlikely to moderate the likelihood of maladaptive outcomes (Cyders, 2013; DeVito et al., 2020), which was also confirmed by our lack of gender by urgency interactions.

Group comparisons indicated a later mid-sleep time in women athletes, which directly contrasts data from non-athlete populations (Roenneberg et al., 2004). However, generalized linear modelling rendered this gender difference to non-significance. Considering the magnitude of effect of the original gender difference (43 min; *d* = 0.59), this suggests that cognitive fitness levels account for a meaningful amount of variance in sleep timing for women. Later sleep timing has been associated with cardiometabolic, psychological, and performance risks (Partonen, 2015; Facer-Childs et al., 2019). Therefore, cognitive fitness may be a potential intervention target for women athletes under chronic stress to reduce their risk of sleeping later, relative to men athletes.

Women athletes in our sample reported poorer mental health symptoms relative to men—in line with a systematic review on this association during the pandemic (Jurecka et al., 2021). We observed this persists even after controlling for cognitive fitness traits. Women athletes being at a greater risk of poor mental health is not unique to the recent pandemic (Schaal et al., 2011; Walton et al., 2021). Our finding emphasizes the need for more tailored mental health support (amongst other resources) for women athletes throughout their career (Rice et al., 2016; Walton et al., 2021).

After controlling for gender, higher self-control predicted lower depression, lower intolerance of uncertainty predicted lower anxiety, and higher premeditation predicted higher total sleep time. These three findings are all in line with previous studies (Miller and Rucas, 2012; Li et al., 2020; Li et al., 2021). However, we extend the literature by reporting these in a group of competitive athletes during the pandemic. Taken together, our data indicate that certain cognitive fitness traits may confer measurable mental health and sleep benefits that hold in competitive sport contexts and under conditions of chronic stress.

In our sample of competitive athletes, lower sensation seeking impulsivity predicted higher depression and stress after controlling for gender. This may be explained by the behavioral activation (Cuijpers et al., 2007) involved in seeking out thrills/challenges associated with competitive sport. This same association has been previously found when using physiological markers of stress (Frenkel et al., 2018), and our finding extends this to psychological stress under chronic conditions.

We found a positive association between premeditation and state anxiety, which may be explained by the tendency for anxious individuals to demonstrate concern regarding future events and thus premeditating consequences (Wells, 1995). Although we cannot disentangle the causal direction of premeditation and anxiety, it is possible that the chronicity of the COVID-19 pandemic may have played a role in exposing this positive relationship within athletes.

The only significant interaction term across all models was gender by perseverance in the prediction of depression such that, for men only, perseverance was associated with increased depression. One possible explanation is that perseverance is manifesting as a maladaptive presentation of perfectionism, which in turn is associated with greater symptoms of low mood (Kannis-Dymand et al., 2020). Our gender-specific finding may speak to the change in lifestyle caused by the pandemic and the potential consequences for men athletes adapting to this specific change using perseverance-related traits.

### 4.1 Methodological considerations

We considered gender differences based on the athletes’ self-reported gender identification and did not assume underlying biological characteristics, which we propose as a strength in the facilitation of clear terminology use (Madsen et al., 2017). However, our dataset precludes us from determining whether identified gender differences may be explained by factors associated with biological sex such as testosterone levels and/or the menstrual cycle (Baker and Driver, 2007; Hines, 2010; Giltay et al., 2012). Where possible, these should be considered in future research.

We did not control for possible gender differences in reporting biases such as social desirability. However, we are unaware of published data suggesting this for our measures in competitive athletes during the pandemic. Encouragingly, no gender difference in social desirability has been found in attitude- and behavior-related items during the pandemic amongst a more general sample (Galasso et al., 2020). Data across all our measures may have been more robust if they were collected objectively or *via* clinical interview, especially in the case of our one-item measures for sleep. Future research should consider these methods when associating cognitive fitness to wellbeing outcomes.

The study aimed to capture a snapshot of how relatively stable traits predicted sleep and mental health at a specific point in time. However, despite the use of trait-based cognitive fitness measures, the study design was cross-sectional, so causality between cognitive fitness and our outcomes cannot be known for certain. Future research may consider a controlled experimental design. Furthermore, despite being under the same chronic stressor, the dynamic effects that the COVID-19 pandemic had across athletes and between measure administration could also not be accounted for. It may thus be worth accounting for this dynamic variability by employing longitudinal designs in future research.

Finally, we acknowledge the relatively modest sample size compared to some (though not all) studies that have investigated sleep and mental health during the pandemic in competitive athletes (see the review by Jurecka et al. (2021)). However, the prior research investigated these outcomes in isolation from cognitive fitness. Thus, our exploratory study is highly novel and strengthened *via* the inclusion of cognitive fitness which exposed the roles of these traits in modulating gender differences and was associated with sleep and mental health. A power analysis was not conducted *a priori* as an effect size in the extant literature was not available given the unique combination of the study’s context (chronic stress), sample, measures, and analyses. It would be beneficial for future studies to aim to replicate our findings in a larger sample that is recruited using probability sampling.

### 4.2 Conclusion and future directions

The ingredients of cognitive fitness studied here were drawn from a theory-driven model (Aidman, 2020) which in turn is based on Research Domain Criteria (Clark et al., 2017) and supported by a transdisciplinary Delphi study (Albertella et al., 2023). The cognitive fitness framework suggests that cognitive fitness factors, although initially trait-based, can be trained for sustained optimal performance (Aidman et al., 2022; Albertella et al., 2023). Women competitive athletes in the current study reported poorer cognitive fitness in multiple domains. Thus, our research suggests that gender differences in cognitive fitness traits should be considered in the creation or application of cognitive fitness training, for example, in program dose or frequency. Our data also indicate, regardless of their level of cognitive fitness, women athletes are more likely to experience poorer mental health. These findings reinforce the calls for more research and tailored support for women athletes (Emmonds et al., 2019; Cowley et al., 2021). Having conducted the study under chronic stress conditions, we also speculate that support may be needed for women athletes during other chronic stressors such as long-term-injuries or post-retirement (Hickey et al., 2016). Finally, in most cases, the associations between cognitive fitness traits and sleep and mental health did not differ between men and women athletes. As future research explores the utility of cognitive fitness training, our findings generate the hypothesis that the performance and mental health of men and women athletes would benefit from training programs targeting cognitive fitness (Aidman et al., 2022).

Together, the study findings support the cognitive fitness framework proposition that cognitive fitness constitutes a protective factor against the accumulation of chronic stress (Aidman, 2020). Despite the study’s limitations, such as subjective biases, the cross-sectional design, and factors associated with biological sex, our findings revealed novel relationships between cognitive fitness, sleep, and mental health, which may inform more targeted interventions for competitive athletes, teams, and sports practitioners.

## Data Availability

The datasets presented in this article are not readily available because public data sharing for this purpose is confidential and ethically restricted. The consent obtained from participants does not extend to making the data publicly available for a third party to use for its own purposes. Requests to access the datasets should be directed to elise.facer-childs@monash.edu.
