# Supplementary material for: Cognitive fitness modulates gender differences in sleep and mental health among competitive athletes under chronic stress
Source: Front Physiol. 2023 Mar 8;14:1118822. doi: 10.3389/fphys.2023.1118822 (PMC10031072; doi:10.3389/fphys.2023.1118822)
Supplement: Supplementary file 1 [file Image1.pdf]

frontiers

Supplementary Material - *Manuscript ID 1118822*

Supplementary Figure 1. Scatterplots with lines of best fit representing the association between each cognitive fitness variable (each row) with each sleep or mental health variable (each column).

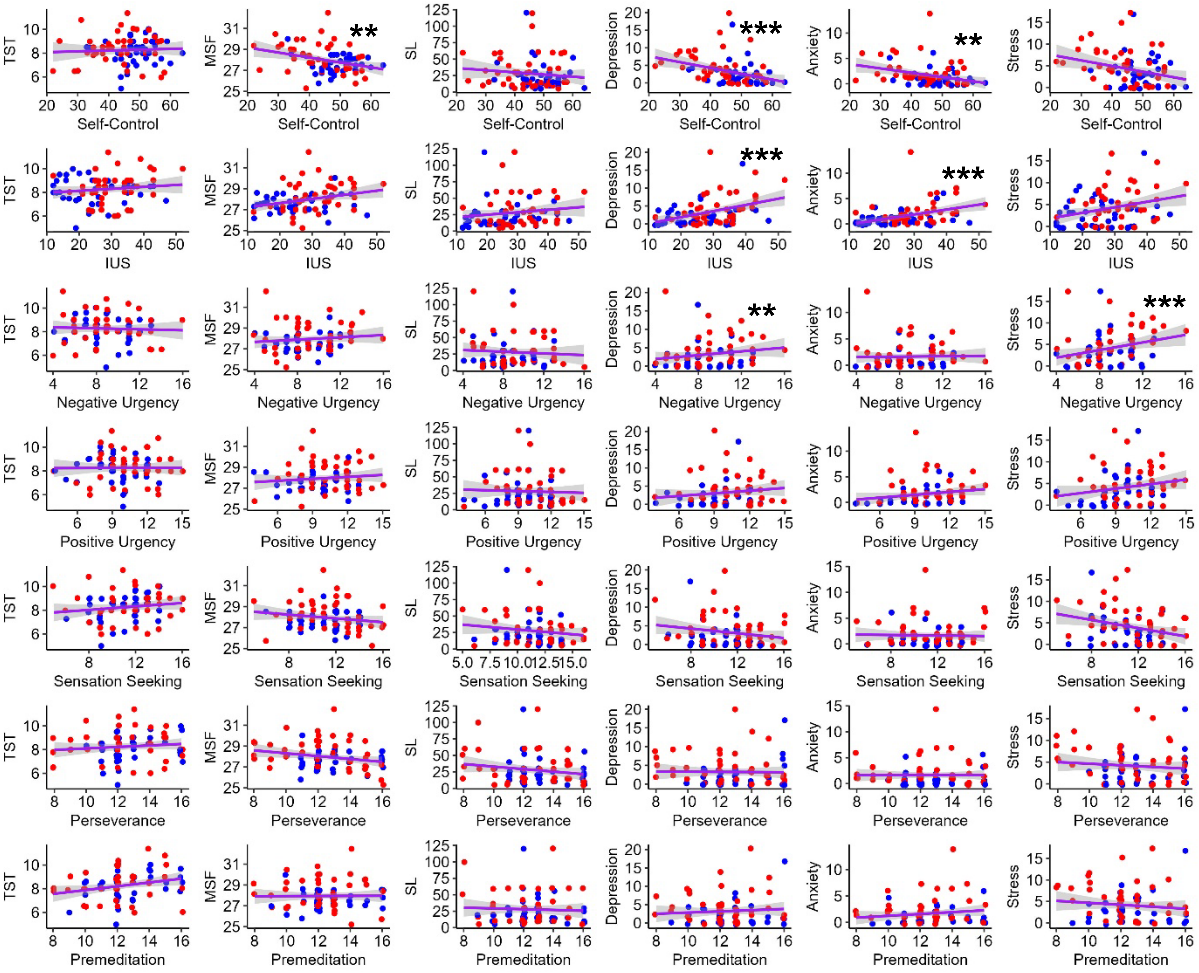

Note. TST = Total sleep time; MSF = Mid-sleep time on free days; SL = Sleep latency; IUS = Intolerance of uncertainty scale. Significant correlations between the x-axis and y-axis variable are indicated in Table 4 of the main text, and those of at least medium effect are indicated here, *$p < .05$; **$p < .01$; ***$p<.001$. Each dot represents a participant. When Supplementary Material is viewed online or in color, the dots are colored by gender: blue = man; red = woman. A linear line of best fit is plotted, with a shaded standard error around the line.
